# Development and validation of the needs of children questionnaire: An instrument to measure children’s self‐reported needs in hospital

**DOI:** 10.1111/jan.14099

**Published:** 2019-07-10

**Authors:** Mandie Foster, Lisa Whitehead, Diana Arabiat

**Affiliations:** ^1^ School of Nursing and Midwifery Edith Cowan Univeristy Joondalup, Perth Western Australia Australia; ^2^ Postgraduate Centre for Nursing Studies University of Otago Christchurch New Zealand; ^3^ The University of Jordan, School of Nursing Amman Jordan

**Keywords:** child health care, child self‐report, children's needs, instrument development, nursing, reliability, validity

## Abstract

**Aim:**

To develop and psychometrically test the needs of children questionnaire (NCQ), a new instrument to measure school‐aged children's self‐reported psychosocial physical and emotional needs in paediatric wards.

**Design:**

This is an instrument development study based on recommendations for developing a reliable and valid questionnaire.

**Method:**

The NCQ was developed over three phases between February 2013–April 2017 and included item generation; content adequacy assessment; questionnaire administration; factor analysis; internal consistency assessment and construct validity. Psychometric properties were assessed after 193 school‐aged children completed the needs of children's questionnaire in four paediatric areas in Australia and New Zealand.

**Results:**

The development and validation of the NCQ over two countries resulted in a 16‐item, four‐category tool to measure the self‐reported importance and fulfilment of school‐aged children's needs in hospital. Cronbach's alpha for the combined samples was 0.93.

**Conclusion:**

The NCQ bridges a gap to measure the level of importance and fulfilment of school‐aged children's self‐reported needs in hospital. Future testing and validation is needed in other paediatric areas and countries.

**Impact:**

The 16‐item NCQ is a valid measure to evaluate if the quality of care delivered and received in hospital is in line with what children self‐report as important and required and to date indicates good usability and utility. Child self‐reports are essential to inform healthcare delivery, policy, research and theory development from a child and family‐centred care lens that honours the United Nations Convention on the Rights of the Child and the best interests of the child.

## Abstract (zh)

**目的:**

编制儿童需求调查问卷并对其进行心理学测试,该问卷是一种新的测量儿科病房学龄儿童自述社会心理、生理、情感需求的量表。

**设计:**

这是一项基于编制可信有效问卷建议进行的量表编制研究。

**方法:**

从2013年2月到2017年4月,儿童需求调查问卷的编制超过三个阶段,包括项目生成、内容适当性评估、问卷管理、因子分析、内部相容性评估以及结构效度等阶段。来自澳大利亚、新西兰四个地区儿科病房的193名学龄儿童填写了儿童需求调查问卷,随后我们对问卷的心理测量特质进行了评估。

**结果:**

儿童需求调查问卷在两个国家进行编制和验证,最终产生了一个16项4范畴的量表用于测量学龄儿童自述住院需求的重要性和完成度。样卷的综合克隆巴赫系数为0.93。

**结论:**

儿童需求调查问卷填补了学龄儿童自述住院需求重要性和完成度等级测量的空白。未来还需要在其他国家和地区的儿科病房测试及验证该问卷。

**影响:**

16项儿童需求调查问卷是一个有效评估医院提供以及儿童接受的看护质量是否与儿童自述重要需求质量一致并确定可用性、有效性高的量表。儿童自述以儿童和家庭为中心,符合《联合国儿童权利公约》以及儿童的最大利益,为医疗的提供、政策、研究以及理论发展提供了相关信息,因而十分重要。

## 1 INTRODUCTION

The needs of children questionnaire (NCQ) is the first questionnaire to measure the importance and fulfilment of school‐aged children's self‐report on their psychosocial physical and emotional needs in hospital which is in line with a child‐centred care (CCC) lens that honours the United Nations Conventions on the Rights of the Child (United Nations General Assembly, 1989) and the Best Interest of the Child (BIC) model (Kalverboer & Zijlstra, 2006).

### 1.1 Background

Family‐centred care (FCC) is an internationally accepted philosophy of care that places families as central to care delivery whereas CCC situates the child and their interests as central to care delivery (Carter, Bray, Dickinson, Edwards, & Ford, 2014; Christian, 2016; Shields, 2015, 2016). Internationally, work is underway to explore how children can be more centrally positioned and a model of child‐ and family‐centred care (CFCC) is under development which places the child as central to care delivery in the context of family and community that involves the inclusion of children, parents and families in shared decision‐making (SDM) (Coyne, Hallstrom, & Soderback, 2016; Livesley & Long, 2013; Nilsson et al., 2013; Shields, 2018). The core principles of FCC and CFCC include dignity and respect, information sharing, partnership, and collaboration (Coyne, O'Neill, Murphy, Costello, & O'Shea, 2011; Insitute for Patient & Family Centred Care, 2017; Sala Institute for Child & Family Centered Care, 2018). There is a plethora of literature on students', parents', and staff's perceptions of FCC theory, education, research and practice in developed and developing countries (Foster, Whitehead, Arabiat, & Frost, 2018; Hill, Knafl, & Santacroce, 2017; Kuo et al., 2012; Shields et al., 2012) with limited literature on the child's self‐reported perceptions of their healthcare needs (Anderson & Dolva, 2015; Carter et al., 2014; Dickinson, Wrapson, & Water, 2014; Rasmussen, Water, & Dickinson, 2017).

Many healthcare charters, committees, and policy documents state that care must be aligned to protect and act in the “best interests of the child” driven by the principles of respect, honesty, information, age appropriate means, and opportunities to freely participate in SDM as forefront to care delivery (Children’s Commissioner, 2010; Lundy, McEvoy, & Byrne, 2011; United Nations Committee on the Rights of the Child, 2013; United Nations General Assembly, 1989; World Health Organization, 1986). Historically, the literature on children's needs and experiences in healthcare settings have largely been limited to qualitative designs or tools completed by adults as proxies for children (Coyne et al., 2016; Dickinson et al., 2014; Gibson, Aldiss, Horstman, Kumpunen, & Richardson, 2010; Rasmussen et al., 2017). Recently there has been an increase in the development of new child self‐report measures (Holder, 2012; Ronan, Dreer, Maurelli, Ronan, & Gerhart, 2014; Unit Developmental and Educational Psychology Institute of Psychology, & Leiden University, 2017) and modification of existing tools to include children and adults' perspectives with children viewed as capable informants of healthcare (Berman, Liu, Ullman, Jadbäck, & Engström, 2016; Orcesi et al., 2014; Rieffe et al., 2016; Toomey et al., 2015; Toupin et al., 2016).

Discourse around how to include the child's perspective directly from the child and their parents as proxies raises methodological, organizational, ethical, and legal challenges (Soderback, Coyne, & Harder, 2011) yet to incorporate the child and parents' right to be heard, respected and involved in evidence‐based health care is needed to gain a holistic socio‐political and familial perspective (Bluebond‐Langner, Belasco, & DeMesquita Wander, 2010; Kalverboer & Zijlstra, 2006; Söderbäck, 2013). Of interest, differences between child's self‐reports and adult reports by proxy have been evident in studies involving children with intellectual disabilities (Goodman, 2001; Haynes, Gilmore, Shochet, Campbell, & Roberts, 2013), Duchenne Muscular Dystrophy (Hendriksen et al., 2017), traumatic brain injuries (Lloyd, Ownsworth, Fleming, & Zimmer‐Gembeck, 2017), neuromuscular disorders (Orcesi et al., 2014), quality of life (Berman et al., 2016), and functional outcomes in paediatric critical care survivors (Ong, Hau Lee, Leow, & Puthucheary, 2016).

Vandenhole, Desmet, Reynaert, and Lembrechts (2017) distinguish between consultative, collaborative, and child‐driven participation where knowledge gained to inform decision‐making is consultative and participation that includes direct involvement in decision‐making is collaborative (Vandenhole et al., 2017). Shier (2001) proposes that irrespective of a child's age the child should be listened to, supported and involved in expressing their views with SDM which is in line with the United Nations Convention on the Rights of the Child (UNCRC) and Roger Hart's 6th, 7th, and 8th steps for children's participation in decision‐making (Shier, 2001; United Nations General Assembly, 1989). Nilsson et al. (2013) and Soderback et al. (2011) reiterate that FCC and CCC need to include a child's perspective (child's view) and child perspective (parent's view), as a relationship exists between the two. Hence the child's, parents', and families' needs, need to be acknowledged and included to facilitate the best evidence‐based practice and health outcome for children and families in hospital as in line with CFCC.

A questionnaire to evaluate if the quality of care delivered and received in hospital is in line with what children self‐report as important and required is needed to maximize positive healthcare experiences and inform healthcare delivery, policy, research, and theory development.

## 2 THE STUDY

### 2.1 Aim

The purpose of this study was to develop and psychometrically test the NCQ, a new instrument to measure school‐aged children's self‐reported psychosocial physical and emotional needs in hospital.

### 2.2 Methodology

The development of the NCQ followed six of the seven stages recommended by Hinkin, Tracey, and Enz (1997) and included item generation; content adequacy assessment; questionnaire administration; factor analysis; internal consistency assessment and construct validity. Confirmatory factor analysis and convergent, discriminant‐ and criterion‐related validity were not assessed due to sample size and the absence of available tools to measure the same or dissimilar construct.

### 2.3 Participants

Parents and children were recruited and invited to participate in the study by the chief investigator, research assistant or clinical nurse specialist from two hospitals in Australia and New Zealand (NZ). Inclusion criteria included signed written parent and child consent/assent, a hospital admission greater than 24 hr, developmental age of the child between 5 and 16 years of age and a good understanding of the English language.

### 2.4 Instrument

#### 2.4.1 Item Generation

The items were initially generated using an inductive thematic approach from a meta‐synthesis of primary research on children's needs in hospital undertaken from 1998 to 2014 (Foster, Whitehead, & Maybee, 2010, 2016; Foster, Whitehead, Maybee, & Cullens, 2013; Shields et al., 2012). Items were selected and classified into codes, categories, and themes based on similarity of meaning (Thomas, 2006).The same items were then deductively classified using the needs of parents' questionnaire (NPQ) (Kristjansdottir, 1995) and BIC model (Kalverboer & Zijlstra, 2006) as a theoretical framework. The items were placed under one of the five NPQ domains (trust, to be trusted, information, support/relationships, and resources/facilities) that correlated with the physical psychosocial and emotional needs of children in hospital (Polit & Beck, 2008). The BIC model includes 14 socio‐familial environmental conditions that influence a child's holistic development (Kalverboer & Zijlstra, 2006) and the NPQ is a 51 statement tool that measures the importance, fulfilment and independence of parents' psychosocial physical and emotional needs in hospital from the staff or parents' perception (Foster & Whitehead, 2017; Shields & Kristensson‐Hallstrom, 2004). Here the NPQ acted as a template for children's needs and the BIC focused on the unique socio‐political familial factors that influenced children's experiences, both adding to the development of a tool that had a CFCC lens. A 3‐point Likert scale was created to measure the degree of perceived importance being “very important”, “important”, and “not important” and on whether that need had been met “happened all the time”, “happened sometimes” or “did not happen”. A higher score indicated greater perceived importance and fulfilment.

All items addressed a single issue and had a Flesch‐Kincaid Score (FKS) of < 3 equivalent to a 3‐year or 7‐year‐olds comprehension and a Flesch‐Kincaid Reading Ease (FKRE) score range from 82 to 117 indicating easier readability (Flesch, 1948). The 3‐year level has been a common benchmark for children's self‐report tools (Deighton et al., 2014). Double negatives and leading or double barrelled questions were avoided (Polit & Beck, 2008; Rattray & Jones, 2007). Additional open‐ended questions were included on six statements (nine items) that had a high importance score being “how can we do this” or “how can we help this happen” as well as the child's age, illness, admission type, days spent in hospital, ethnicity and use of the NCQ (Creswell & Clark, 2011). All the open‐ended responses underwent thematic and critical analyses to provide guidance on the iterative development of the tool, subsequent revisions, and pilot studies.

#### 2.4.2 Content Adequacy Assessment

**Figure 1:**
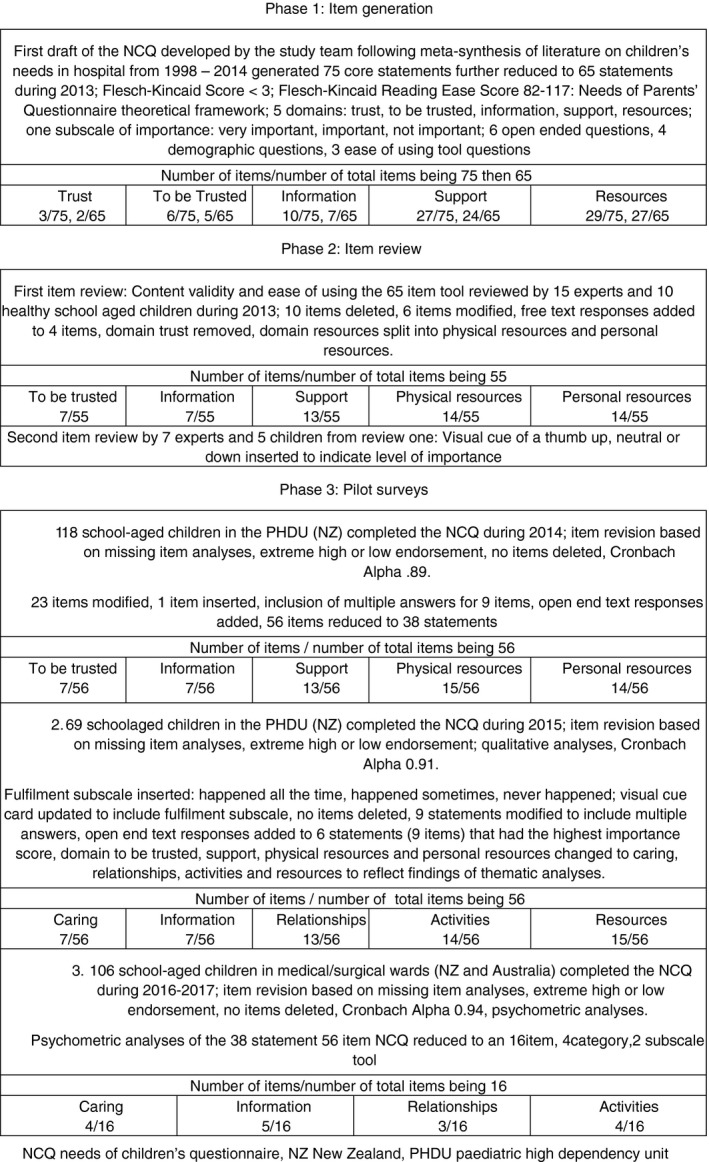
Development of the needs of children's questionnaire

Construct validity, item deletion and modification of the NCQ were assessed with different samples over three phases (Figure 1). Phase one (2013)—item review, face, and content validity of the initial 65 statement tool were critiqued by 15 purposively recruited international, national, and local multidisciplinary paediatric experts for clarity, relevance, word use, appropriateness, and recommendations by completing a critiquing template. Ease of answering the 65‐item tool, use of the 3‐point importance Likert scale, understanding the content and recommendations were undertaken by 10 purposively recruited healthy school‐aged children in NZ who had experienced a prior hospital admission. Phase two included a second review where seven experts and five children from phase one provided feedback on the revised 55 item tool on whether the items, domains and concepts of children's needs in hospital were covered.

Phase three included three pilot studies (Figure 1). Study 1 (2014)—ease of answering the 55‐item tool, use of the 3‐point importance Likert scale, understanding the content and checking for response errors were undertaken by 18 purposively recruited hospitalized school‐aged children in a paediatric high‐dependency unit (PHDU) in NZ. In addition open‐ended questions were used to gain a deeper understanding on how children of various ages interpreted and responded to each item (Creswell & Clark, 2011). Items were deleted in the case of extreme high or low endorsement and new items inserted or modified (Rattray & Jones, 2007). Study 2 (2015)—ease of answering the 38 statement (56 item) tool, use of the 3‐point importance Likert scale, understanding the content and recommendations were undertaken by 69 conveniently sampled hospitalized school‐aged children in a PHDU in NZ. Thematic analyses were undertaken on all open‐ended responses to ensure all needs were represented. Study 3 (2016–2017)—ease of answering the 38 statement (56 item tool), use of the 3‐point importance and fulfilment Likert scale, understanding the content, recommendations and open text sections were undertaken by 106 conveniently sampled hospitalized school‐aged children in three paediatric medical and surgical wards in Australia and NZ.

#### 2.4.3 Questionnaire Administration

The retained items, modified versions, recruitment strategies, logistics, and level of burden in completing the tool were assessed for different samples using purposive and convenience sampling at every stage. A picture of a hand with the thumb up (very important, happened all the time), thumb neutral (sort of important, happened sometimes), and thumb down (not important, did not happen) was used as a visual cue to illustrate the different levels of importance and fulfilment. The combined sample size of 193 children who completed the tool was adequate to evaluate the item to response ratio for the level of importance (*N* = 193), fulfilment (*N* = 106) and domains (*N* = 193).

#### 2.4.4 Statistical analysis: factor analysis

To evaluate the factor structure of the 56‐item NCQ, an exploratory factor analysis (EFA) was conducted. Another reason for using the EFA was to reduce the set of 56 items to a shorter modified version (possibly 10–15 items that children could easily complete during their hospital stay) that clearly represented the content of the underlying constructs (Hinkin et al., 1997). Prior to performing the factor analysis, the Kaiser‐Meyer‐Olkin (KMO), a measure of sampling adequacy test, was performed to evaluate data suitability for factor analysis (Kaiser, 1974). The KMO test was 0.68 with the Bartlett test of sphericity being significant (χ2 = 555.55, *df* = 153, *p* ≤ 0.001) indicating that there were significant relationships to investigate (Tobias & Carlson, 1969).

The original 56‐item NCQ did not hold a pre‐determined assumption that specified an appropriate number of expected subscales, or where each item should hypothetically belong. Statistically, there were minimum variations between children's ratings; therefore, as Hinkin et al. (1997) recommended, an EFA using principal axis factor analysis was undertaken to account for error variances, improve the model fit and reduce the number of items (Ford, MacCallum, & Tait, 1986; Rummel, 1970). For this study, an item was retained where an item: exhibited a loading >0.40 (Kline, 2011); (2) did not cross‐load (Tabachnick & Fidell, 2013); and demonstrated uniqueness <0.80. A minimum of three items per factor were considered in the analysis and attention to both psychometric quality and meaning of interpretation applied. Items that did not meet these criteria were sequentially removed one by one. Further analyses determined on the modified 16 item NCQ included measures of internal consistency using Cronbach's alpha coefficient (Cronbach, 1951; Kumar, 2015) for the individual samples (*N* = 18, NZ; *N* = 69, NZ; *N* = 59, NZ; *N* = 47, Australia) and inter‐item correlations for the combined sample (*N* = 193) (Nunnally & Bernstein, 1994; Streiner & Kottner, 2014).

### 2.5 Ethical considerations

This study was approved by the ethics committees, universities and hospitals in Australia and NZ and honoured the ethical principles of informed consent, respect, beneficence, and confidentiality.

## 3 RESULTS

### 3.1 Item generation and review

A graphical illustration of the development process for the 16‐item NCQ is displayed in Figure 1. A meta‐synthesis of the literature generated an initial 75 items further synthesized to 65 and 55 core items after a critical review of the items by 15 of 32 (47% response) paediatric experts and all (10, 100% response) healthy school‐aged children. The items were placed into five domains and were similar to those items parents perceived as important and needed in hospital albeit from a child's perspective (to be trusted, information, support, personal resources, physical resources). A picture of a thumb being up (very important), neutral (somewhat important) and down (not important) were added as a visual cue to indicate a level of importance (Figure 1).

### 3.2 Pilot studies

The demographic characteristics of all participants included in the pilot studies are displayed in Table 1. Study 1 included 18 school‐aged children who completed the 55 item NCQ in a PHDU in NZ. All the admissions were unplanned (*N* = 18, 100%) with most children between 11–15 years of age (*N* = 8, 44%) of European ethnicity (*N* = 11, 65%) and a hospital stay <2 days (*N* = 10, 56%) (Table 1). Fifty‐six items were synthesized into 38 statements, no items were deleted, open text responses were added and the item “to get back to school” was inserted (Figure 1). All the children stated they understood the questions and 17 children liked using the ipad. The total importance mean score (TIMS) was 122.4 (*SD* 13.12) (range 97–160) and an alpha coefficient of 0.89.

**Table 1:** Pilot studies: children's scores and demographic variables

Variable	Study 1	Study 2	Study 3	
Country	New Zealand	New Zealand	New Zealand	Australia
Setting	PHDU (1)	PHDU (1)	Medical/Surgical (2)	Medical/Surgical (1)
Sample	*N* = 18	*N* = 69	*N* = 59	*N* = 47
Admission type				
Planned	*N* = 0, (0%)	*N* = 10, (15%)	*N* = 9, (15%)	*N* = 17, (36%)
Unplanned	*N* = 18, (100%)	*N* = 59, (75%)	*N* = 50, (85%)	*N* = 30, (64%)
Total mean score				
Importance	122.4 (*SD* 13.12)	136.43 (*SD* 14.17)	134.52 (*SD* 12.66)	125.00 (*SD* 16.75)
Range	97–160	98–161	107–164	95–160
Fulfilment			121.37 (*SD* 15.88)	122.00 (*SD* 15.58)
Range			64–162	88–155
Cronbachs alpha		0.890	0.910	0.944
Length of stay				
1–2 days	*N* = 10, 56%	*N* = 48, 71%	*N* = 40, 68%	*N* = 30, 64%
3–4 days	*N* = 5, 28%	*N* = 11, 16%	*N* = 11, 19%	*N* = 15, 32%
5–7 days	*N* = 1, 6%	*N* = 2, 3%	*N* = 4, 7%	*N* = 2, 4%
>7 days	*N* = 2, 11%	*N* = 7, 10%	*N* = 4, 6%	*N* = 0, 0%
Age				
5–7 years	*N* = 6, 33%	*N* = 18, 26%	*N* = 11, 19%	*N* = 10, 22%
8–10 years	*N* = 4, 22%	*N* = 22, 32%	*N* = 16, 27%	*N* = 7, 15%
11–15 years	*N* = 8, 44%	*N* = 29, 42%	*N* = 32, 54%	*N* = 30, 63%
Use of the NCQ				
Understood questions	*N* = 18, 100%	*N* = 69, 100%	*N* = 59, 100%	*N* = 45, 96%
Liked using the ipad	*N* = 17, 95%	*N* = 65, 95%	*N* = 57, 97%	*N* = 43, 92%
Ethnicity				
European	*N* = 11, 65%	*N* = 46, 67%	*N* = 36, 61%	*N* = 36, 77%
Māori, Aboriginal	*N* = 2, 12% (M)	*N* = 14, 20% (M)	*N* = 13, 22% (M)	*N* = 3, 6% (A)

Abbreviations: A, Aboriginal/Torres Strait Islanders; PHDU, paediatric high‐dependency unit; M, Maori; NCQ, needs of children questionnaire.

Study 2 included 69 school‐aged children who completed the 38 statement 56‐item NCQ in a PHDU in NZ. Fifty‐nine of the admissions were unplanned (86%) with most children between 11 and 15 years of age (*N* = 29, 42%) of European ethnicity (*N* = 46, 67%) with a hospital stay < 2 days (*N* = 48, 71%) (Table 1). The domains to be trusted, support, personal resources and physical resources were changed to caring, relationships, activities and resources to reflect the thematic analyses of the open‐ended responses. Thematic analyses included 265 verbal and 27‐typed responses synthesized into nine themes (coping strategies, getting better, family, environment, treatment, relationships, facilities, food, and visitors) and two syntheses (priorities and choices). Activities included resources for the child indicative of a CCC lens and resources included facilities for the parents and/or family reflecting a FCC lens. A fulfilment subscale was inserted to measure the extent to which a need was met, no items were deleted, and open‐end text responses were added to six statements that had the highest importance score (Figure 1). All the children stated that they understood the questions and 65 children liked using the ipad. The TIMS was 136.43 (*SD* 14.17) (range 98–161) and an alpha coefficient of 0.91.

Study 3 included 106 school‐aged children in three medical and surgical wards in NZ and Australia who completed the 38 statement 56‐tem, five‐category tool. Most children (NZ *N* = 59; Australia *N* = 47) were between 11 and 15 years of age (*N* = 32, 54%; *N* = 30, 63%) of European ethnicity (*N* = 36, 61%; *N* = 36, 77%) with a hospital stay <2 days (*N* = 40, 68%; *N* = 30, 64%) (Table 1). In NZ, the TIMS 134.52 (*SD* 12.66) (range 107–164), total fulfilment mean score (TFMS) 121.37 (*SD* 15.88) (range 64–162) and alpha coefficient of 0.91 were similar to the Australian TIMS 125 (*SD* 16.75) (range 95–160), TFMS 122 (*SD* 15.58) (range 88–155) and an alpha coefficient of 0.94 (Figure 1, Table 1).

### 3.3 Psychometric testing

In this study, EFA procedures were used to assess the underlying dimensions of the 56 items comprising the NCQ tool (Hinkin et al., 1997). Principal axis principal component analysis (PCA) with varimax rotation was used in the factor analyses. Principles used to determine how many factors to retain included Kaiser's criterion (Field, 2018), parallel analysis (Abdi & Williams, 2010; Horn, 1965; Zwick & Velicer, 1986) and examination of the scree plot (Cattell, 1966; Ledesma, Valero‐Mora, & Macbeth, 2015).

**Figure 2:**
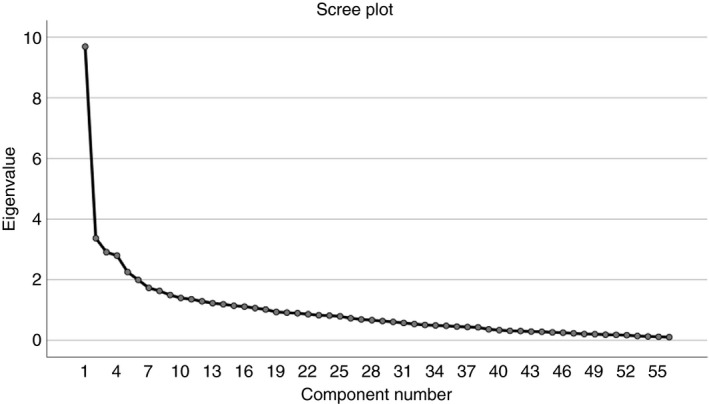
Slope of scree plot

In this study, a PCA identified 18 factors with eigenvalues >1.0. In consecutive order, eigenvalues for the first 18 components were 9.68, 3.36, 2.90, 2.79, 2.25, 1.99, 1.72, 1.62, 1.49, 1.39, 1.35, 1.28, 1.22, 1.18, 1.14, 1.11, 1.06, and 1.01. The results of the parallel analysis suggested six factors where real‐data eigenvalues exceeded random‐data eigenvalues. The eigenvalues (and % of variance accounted for) were 9.68 (2.55%), 3.36 (2.37%), 2.9 (2.25%), 2.79 (2.14%), 2.25 (2.05%), and 1.99 (1.98). The number of factors to extract were based on a parallel analysis of 1,000 datasets, using the 95% cut‐off (O’Connor, 2000) and indicated retention of six factors. A decision was made to examine the scree plot to get a sense of the pattern of factor coefficients for the 56 items of the measure (Figure 2).

Since the parallel analysis suggested a six‐factor solution might also be plausible, this solution was explored through EFA. An examination of the pattern of factor coefficients revealed 25 items with multiple loadings in the rotated factor solution and communality values <0.2 and/or factor loadings <0.4 (activities *N* = 7, information *N* = 1, relationships *N* = 6, caring *N* = 2, resources *N* = 9). At the same time, 13 items were deleted because uniqueness was >0.80 (activities *N* = 3, information *N* = 1, relationships *N* = 4, caring *N* = 1, resources *N* = 4). After removing these items, an additional five‐factor EFA solution was generated, with a clearer factor structure pattern beginning to emerge. While the parallel analysis findings suggested six components, yet a 5‐factor solution yielded a better structure with lower correlation between factors.

For the five‐factor solution four items loaded onto factor one: activities (ACT, activities); five items onto factor two: information (INF, information); three items onto factor three: relationships (REL, relationships), four items onto factor four: caring (CAR, caring) and two items onto factor five: resources (RES, resources) (Table 2). The fifth factor resources was excluded due to the low number of loaded items (*N* = 2) and likelihood for low reliability and replication in future studies (Field, 2018).

**Table 2:** Exploratory factor analysis: pattern matrix

Factors and items	Factor 1	Factor 2	Factor 3	Factor 4	Factor 5
ACT4: To be able to go to the playroom	**0.744**	−0.082	0.090	0.174	0.266
ACT2: To have special treats after a test (presents)	**0.725**	0.000	0.208	0.057	0.226
ACT3: To be able to do arts and crafts	**0.733**	0.313	0.124	0.096	0.087
ACT1: To have books to read	**0.692**	0.424	0.046	0.084	0.022
INF1: That staff tell me the medicines I'm having	0.166	**0.709**	−0.009	−0.035	0.131
INF2: That staff tell me my test results	0.085	**0.642**	0.166	0.006	0.120
INF5: To get back to school	0.296	**0.604**	−0.181	0.392	−0.244
INF4: To have staff show me how the machines work	0.370	**0.505**	0.246	−0.022	0.064
INF3: To talk about how my illness may affect me	−0.076	**0.541**	0.181	0.331	−0.246
REL3: That staff listen to me	0.200	0.099	**0.681**	0.229	0.035
REL1: That I choose when I have visitors (family/friends)	0.048	−0.015	**0.648**	−0.112	0.320
REL2: To have the same nurse or doctor care for me	0.159	0.248	**0.634**	−0.045	−0.184
CAR3: To feel the staff care about me	0.164	−0.091	0.397	**0.630**	0.305
CAR4: To have mum, dad or my family help care for me	0.288	0.182	−0.205	**0.625**	0.226
CAR1: To know I am safe and well looked after	−0.160	0.071	−0.137	**0.597**	−0.064
CAR2: To not see other children sad or upset	0.238	−0.173	0.287	**0.507**	0.137
RES1: To have places my parents or family can go to have a shower	0.344	0.124	0.086	0.209	**0.799**
RES2: To have places my parents or family can go to get food or drink	0.148	0.045	0.084	0.134	**0.777**

Abbreviations: ACT, activities; CAR, caring; INF, information; REL, relationships; RES, resources.

The final NCQ included four factors and 16 items with reliability scores of 0.74 (ACT), 0.58 (INF), 0.47 (REL) and 0.41 (CAR) respectively with 0.69 for the total scale. The distribution of items in these four factors for the combined sample is presented in Table 3.

**Table 3:** Inter‐item correlation matrix

Correlation	ACT 1	ACT 2	ACT 3	ACT4	INF 1	INF 2	INF 3	INF 4	INF 5	REL1	REL2	REL3	CAR1	CAR2	CAR3	CAR4
ACT1	1.00	0.33	0.49	0.35	0.30	0.16	0.05	0.25	0.30	0.09	0.12	0.15	−0.04	0.05	0.01	0.16
ACT2		1.00	0.44	0.43	0.05	0.06	−0.01	0.22	0.01	0.14	0.07	0.13	−0.12	0.21	0.09	0.14
ACT3			1.00	0.35	0.16	0.16	0.06	0.23	0.22	0.05	0.20	0.13	−0.05	0.00	0.13	0.13
ACT4				1.00	0.04	−0.06	−0.17	0.21	0.10	0.05	0.02	0.13	−0.06	0.18	0.22	0.18
INF1					1.00	0.40	0.19	0.31	0.23	0.08	0.08	0.04	0.01	−0.07	−0.03	0.09
INF2						1.00	0.11	0.32	0.24	0.40	0.09	0.19	−0.01	−0.02	0.07	0.09
INF3							1.00	0.18	0.24	0.11	0.21	0.13	−0.00	0.00	0.08	0.11
INF4								1.00	0.15	0.32	0.18	0.14	0.10	0.08	0.01	0.04
INF5									1.00	0.24	0.14	0.02	0.10	0.06	0.02	0.24
REL1										1.00	0.27	0.21	0.01	0.06	0.13	−0.07
REL2											1.00	0.27	−0.13	0.04	0.06	−0.09
REL3												1.00	−0.05	0.17	0.31	0.04
CAR1													1.00	0.11	0.21	0.04
CAR2														1.00	0.29	0.15
CAR3															1.00	0.24
CAR4																1.00

Abbreviations: ACT, activities; CAR, caring; INF, information; REL, relationships.

## 4 DISCUSSION

To our knowledge, no instrument is available to assess the perception of need of school‐aged children during a hospital stay. Therefore, this study builds on the state of the science on the CCC literature to enable a better understanding of children's self‐reported needs in hospital. It is essential to evaluate and drive care delivery to align with the areas that children report as important and promote children's participation as active research participants in healthcare directives. In this regard, the NCQ is a new valid tool to measure the school‐aged child's self‐reported needs in hospital.

On reviewing the current literature for child self‐report measures, since the initial process of developing the NCQ, the scales and sub‐scales on social relationships, school, family functioning, cognitive thoughts, behaviour, depression, anxiety, self‐care, and sensory experiences were evident across many of the measures for children living with chronic illness with a significant gap between the needs of children experiencing acute health changes and needs in the hospital setting (Deighton et al., 2014; Foster, Whitehead, & Maybee, 2016; Ong et al., 2016; Wolpert et al., 2012). The Child Hospital Consumer Assessment of Healthcare Providers and Systems (HCAHPS) is the latest tool to measure a child's hospital experience by parent proxy, in the area of communication, safety, comfort, environment, and global rating (Toomey et al., 2015).

The Child HCAHPS reports to focus on the child and parents' inpatient care with an aim to inform practice, care delivery, health plans, insurers and policy makers, yet a significant limitation in this measure is the absence of the child's perspective (Agency for Healthcare Research & Quality, 2018). With the emergent debate on CFCC and changes in policy to include children as active research participants in healthcare directives, it is evident that the NCQ will build on children's rights to be heard, valued and actively participate in the “best interests of the child” from a child's perspective.

The NCQ (16‐item) measured children's psychosocial physical and emotional needs in four domains of caring, information, relationships and activities on a level of importance and fulfilment as derived from an extensive literature review, consultation and pilot testing with multidisciplinary paediatric experts; healthy and hospitalized school‐aged children in various paediatric general and critical care settings over two countries (Rattray & Jones, 2007). This was to ensure the self‐reported lived needs of children in hospital of various ages, illnesses, gender and ethnicity were included (Cleaver, Walker, & Meadows, 2004). It relied on a broad holistic perspective on the “best interests of the child” and school‐aged children's needs in hospital, based on the child's lived experience. Although the NCQ used the NPQ and BIC as a theoretical framework, familiarity with this model and measure are not a pre‐requisite for using the NCQ.

Overall, the evidence to support the internal consistency of the NCQ and its sub‐scales is promising. The high Cronbach's alpha values and intra‐class correlations indicated homogeneity and reliability of a multidimensional four factor (16 item) instrument with good measurement properties and explained variance (Table 2) (Field, 2018). We aimed to develop a brief measure that children could easily complete as part of their hospital stay that still had enough sensitivity to measure what it was supposed to measure. Hence, items were sequentially removed if factor loadings were <0.30, uniqueness greater than 0.80, items loaded onto more than one factor and a minimum of three items were required to represent each factor (Hinkin et al., 1997). The factor loading scores were acceptable and sample adequacy to perform factor analysis was confirmed by the KMO and Bartlett's test of sphericity (Table 3) (Tabachnick & Fidell, 2007).

In this study, the Cronbach's alpha internal consistency range was wide (0.41–0.74) with a lack of confirmatory factor, convergent, divergent, and test–retest analyses yet the EFA were reported as satisfactory (Streiner & Kottner, 2014). Similarly, the Achenbach System of Empirically Based Assessment (ASEBA) youth response measure (11–18 years) also reported a wide Cronbach's alpha internal consistency range (0.55–0.96) due in part to respondent error, sample variance, item ambiguity, irrelevance or heterogeneity with satisfactory convergent, divergent, and test–retest findings (Deighton et al., 2014; McCrae, Kurtz, Yamagata, & Terracciano, 2011).

It is noted in the literature that children's needs are synergistically interconnected to their parents' needs which was evident in this study when children reported on their parents' needs as being important in the factor “resources”; however, it is important to state that the newly developed NCQ (16‐item) is a tool to measure CCC and not an adaptation of the NPQ for children (Foster & Whitehead, 2018; Nilsson et al., 2013; Soderback et al., 2011). When using the NCQ, the authors recommend to explore for any relationship between the NCQ importance and fulfilment scores, as a need scored as important and not met may predict a child's hospital experience and health outcome more than demography or illness severity (Manning, Hemingway, & Redsell, 2017, 2018).

In this study, most children had an acute illness and short hospital stay (1–2 days) (Table 1) whereas most published child self‐report measures focused on children with chronic illnesses where validity reported on clinical versus normative groups and test–retest reliability ranged from 1 to 24 weeks. Collecting data to inform the test–retest reliability in this study proved unfeasible with short hospital admissions and ethical requirements of children and parents needing to be given at least 24 hr to consider participation. Of interest, Deighton et al. (2014) and Ong et al. (2016) critiqued the psychometric properties of 14 child self‐report measures where nine tools included a parent, teacher and/or staff version as an adjunct to the child's response, the scales ranged from 2‐105 items with 3‐ to 6‐point Likert scales and were available in up to 80 languages.

The literature reports the most appropriate period for children to complete a self‐report measure is between 5‐30 min, which was evident in the NCQ (56‐item) tool. In developing the NCQ, there was a need to balance comprehensiveness and ease of administration with the developmental and physical ability of the child. The NCQ took 10–15 min to complete when self‐administered by adolescents and 15–20 min when administered during an interview with the younger child (5–10 years). During the interview, the statements were read slowly to the child and the child responded by way of sign language, verbal communication and/or by independently using the iPad/electronic device. These strategies are similarly reported in other studies where reading the question, visual cues, technology, cards, and play/art based techniques were used with younger children (Coad, 2007; Driessnack & Furukawa, 2012; Haynes et al., 2013).

Future research recommendations include confirmatory analyses to assess the quality of the factor structure by statistically testing the significance of the overall model and relationships among the items and scales (goodness of fit) with a new sample (>200) (Hinkin et al., 1997; Streiner & Kottner, 2014). There is no set criteria for reporting on a model fit, yet it is recommended to test and report on a variety of indices to reflect the various aspects of the model that are most insensitive to sample size, model misspecification and parameter estimates (Crowley & Fan, 1997; Statistics Solutions, 2018; Tabachnick & Fidell, 2007). From here, to modify, translate, and pilot the NCQ over time to include different versions for specific settings, perceptions, populations, and countries with confirmatory, divergent, convergent, and test–retest analyses to inform the state of the science on school‐aged children's self‐reported psychosocial physical and emotional needs in hospital is required.

The NCQ has several potential applications for healthcare settings moving towards a CFCC model. The NCQ could be used as an internationally recognized audit tool in various healthcare settings to inform practice (care delivery, staff awareness, design and resources), theory (CCC, BIC), education (children, parents, family, staff), research (parent–staff versions) and law (policy) to instigate change and/or support best evidence‐based practice as required to fulfil the UNCRC on the “best interests of the child” from a child and child's perspective.

### 4.1 Limitations

The potential effects of the small effect size on the model interpretation are a major limitation in this study. The standard error of loadings can be larger when the sample size is small (de Winter, Dodou, & Wieringa, 2009). This can generate model error and have an impact on factor recovery, lead to bloated‐specific factors, obscuring the presence of more important factors or distribution of minor factors, so further investigations with a better sample size will help address these issues (Sapnas & Zeller, 2002; de Winter et al., 2009). The reliability score for three of the four factors and total instrument (0.69) were below the recommended minimum (0.70) for research instruments which could be due to the 3‐item response scale and small number of items per factor, items and concepts were not analysed for confirmatory factor analysis or compared for concurrent or discriminant validity with other published paediatric measures, as no such measures exist, as was a test–retest measure for reliability as most children being acute admissions were discharged before day 3. Sensitivity to change, children younger than 5 years, parent and staff perspectives, effect on the child's future well‐being have an impact on service delivery, staff awareness of CCC concepts or differential performance in different ethnic, socio‐economic or healthcare structures were not tested which leaves room for further development and testing globally. The literature states the use of a 3‐point Likert scale can reduce the reliability scores and limit the variability in data (floor or ceiling effect) with decreased sensitivity to change or impact over time. These areas require further consideration in future studies.

## 5 CONCLUSION

The NCQ (16‐item) is the first questionnaire to measure the importance and fulfilment of school‐aged children's self‐report on their psychosocial physical and emotional needs in hospital and to date indicates good usability and utility. Further psychometric testing of the NCQ is needed in various healthcare settings.

## CONFLICT OF INTEREST

None of the authors have a conflict of interest with respect to the authorship and or publication of this article.

## AUTHOR CONTRIBUTIONS

All the authors made substantial contributions to the conception and design, or acquisition of data, or analysis and interpretation of data; they involved in drafting the manuscript or revising it critically for important intellectual content; gave final approval of the version to be published. Each author should have participated sufficiently in the work to take public responsibility for appropriate portions of the content; agreed to be accountable for all aspects of the work in ensuring that questions related to the accuracy or integrity of any part of the work are appropriately investigated and resolved.
